# Antifungal Activity of *Ramulus cinnamomi* Explored by ^1^H-NMR Based Metabolomics Approach

**DOI:** 10.3390/molecules22122237

**Published:** 2017-12-15

**Authors:** Chunpeng Wan, Pei Li, Chuying Chen, Xuan Peng, Mingxi Li, Ming Chen, Junsong Wang, Jinyin Chen

**Affiliations:** 1 Jiangxi Key Laboratory for Postharvest Technology and Nondestructive Testing of Fruits & Vegetables, Collaborative Innovation Center of Post-Harvest Key Technology and Quality Safety of Fruits and Vegetables, College of Agronomy, Jiangxi Agricultural University, Nanchang 330045, China; chunpengwan@jxau.edu.cn (C.W.); ccy0728@126.com (C.C.); liming.xi@hotmail.com (M.L.); chenming@jxau.edu.cn (M.C.); 2 State key laboratory of Natural Medicines, Department of Natural Medicinal Chemistry, Pharmaceutical University, 24 Tong Jia Xiang, Nanjing 210009, China; lipei910425@126.com; 3 Pingxiang University, Pingxiang 337055, China; pengx1104@163.com; 4 Center for Molecular Metabolism, Nanjing University of Science and Technology, 200 Xiao Ling Wei, Nanjing 210014, China

**Keywords:** metabolomics analysis, *Penicillium italicum*, antifungal activity, cinnamaldehyde, *Ramulus cinnamomi*

## Abstract

A ^1^H nuclear magnetic resonance (NMR)-based approach to metabolomics combined bioassay was used to elucidate the antifungal activity of cinnamaldehyde (the main active compound of *Ramulus cinnamomi*) isolated from *Ramulus cinnamomi* (RC). Orthogonal signal correction partial least-squares discriminant analysis (OSC-PLS-DA) of NMR data was constructed to analyze all the *P. italicum* data acquired from the control and treatment groups at 4, 8, and 12 h. Metabolic profiles disclosed metabolic changes that were related to the antifungal effects of cinnamaldehyde against *P. italicum* including oxidative stress, disorder of energy metabolism, amino acids, and nucleic acids metabolism in treatment group. This integrated metabolomics approach provided an effective way to detect the antifungal effects of cinnamaldehyde against *P. italicum* dynamically.

## 1. Introduction

Postharvest diseases are a major factor that cause the decay of citrus fruits during storage. More than 20 postharvest diseases occurred by pathogens infections. There are two categories of citrus postharvest diseases based on the methods of pathogens infections, including preharvest infection diseases and postharvest infection diseases [1]. For postharvest infection diseases, blue mold, green mold and sour rot are the major postharvest diseases caused by *Penicillium italicum*, *Penicillium digitatum*, and *Geotrichum candidum* Link, respectively. On the other hand, for preharvest infections diseases, anthracnose, *Alternaria* rot, and brown rot are the major preharvest diseases caused by *Colletotrichum gloeosporioides* (Penz.), *Alternaria citri*, and *Phytophthora* spp., respectively.

NMR is a very efficient and robust technique for generating metabolic profiling. NMR based metabolomics is capable of identifying and discriminating a large number of differential metabolites and providing valuable information for the understanding of pathological changes and diseases.

In order to control citrus postharvest diseases, some medicinal plants were screened for their antifungal activities against pathogens [2]. *Ramulus cinnamomi* and *Cortex cinnamomi* were selected for their promising antifungal activities [3]. Moreover, *Ramulus cinnamomi* showed good blue mold resistance and defense system in Xinyu tangerine [4]. The two *Cinnamomi* plants showed antibacterial and antioxidant properties [5], anti-gastric [6], anti-influenza virus [7], and vasorelaxation activities [8]. However, to the best of our knowledge, there is no report on the antifungal activities of *Ramulus cinnamomi*. Our previous studies have extracted, isolated, and identified some antifungal compounds from the selected antifungal medicinal plants [9,10], which showed good postharvest diseases resistance and maintenance of fruit qualities [11,12]. In continuation, the purpose of the current study was to elucidate the antifungal compounds of *Ramulus cinnamomi* (RC) and study its antifungal mechanism based on the ^1^H-NMR based metabolomics analysis.

## 2. Results and Discussion

### 2.1. Structure of Compounds ***1***–***2***

The structures of the isolated compounds (**1**–**2**) were elucidated by ^1^H-NMR and ^13^C-NMR. They were identified as cinnamic acid (**1**) and cinnamaldehyde (**2**), respectively [13].

Compound **1**, white power, UV λmax (MeOH) nm: 223, 278. ^1^H-NMR (CDCl_3_, 400 MHz) δ: 6.46 (1H, d, *J* = 15.96 Hz, H-3), 7.40 (3H, m, H-6, 7, 8), 7.56 (2H, m, H-5, 9), 7.81 (1H, d, *J* = 15.96 Hz, H-2). ^13^C-NMR (CDCl_3_, 100 MHz): 128.4 (C-5, C-9), 117.3 (C-7), 129.0 (C-6, C-8), 130.8 (C-4), 134.1 (C-3), 147.2 (C-2), 172.6 (C-1).

Compound **2**, yellow oily liquid, UV λmax (MeOH) nm: 225, 289. ^1^H-NMR (CDCl_3_, 400 MHz) δ: 6.71 (1H, dd, *J* = 7.68, 15.96 Hz, H-2), 7.41-7.56 (6H, m, H-3, 5, 6, 7, 8, 9), 9.69 (1H, d, *J* = 7.68 Hz, H-1). ^13^C-NMR (CDCl_3_, 100 MHz): 128.7 (C-5, C-9), 128.6 (C-7), 129.1 (C-6, C-8), 131.3 (C-4), 134.0 (C-3), 152.9 (C-2), 193.8 (C-1)

### 2.2. Antifungal Activities of RC Extracts and Fractions

**Figure 1:**
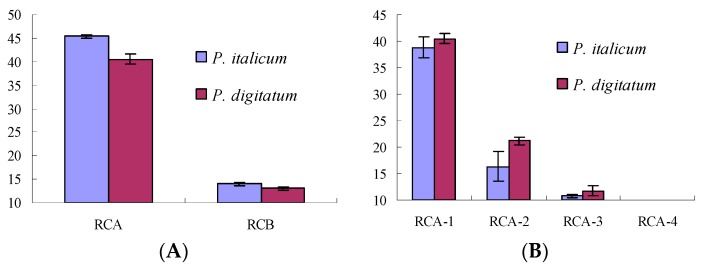
Antifungal activities of RC extracts (**A**) and its fractions (**B**) against *P. italicum* and *P. digitatum.*

RC extracts showed antifungal activity with the DIZs (diameters of inhibition zones) of 34.57 ± 0.78 mm and 31.56 ± 0.66 mm against *P. italicum* and *P. digitatum* at 1.0 g dry materials/mL, respectively. While RCA extracts (extraction by ethyl acetate) showed antifungal activity with the DIZs of 45.33 ± 0.31 mm and 40.53 ± 0.97 mm against *P. italicum* and *P. digitatum* at 50.0 mg/mL, respectively, better (*p* < 0.01, Figure 1A) than that of RCB (extraction by *n*-buthanol, 13.97 ± 0.35 and 13.00 ± 0.30, respectively) at the same concentrations.

The RCA extract was fractionated into four fractions (RCA-1‒RCA-4) by silica gel column chromatography. The antifungal activities of those fractions were tested with results shown in Figure 1B. RCA-1 showed stronger antifungal activity than that of other fractions (*p* < 0.01), and was further investigated for antifungal constituents. 

### 2.3. Antifungal Activity of Cinnamaldehyde

**Figure 2:**
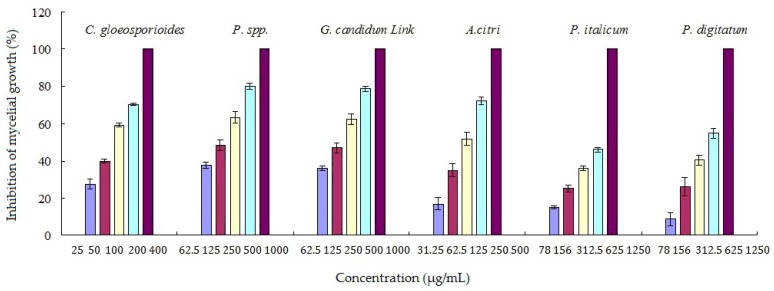
Antifungal activities of cinnamaldehyde against six kinds of citrus pathogens.

The antifungal activities of the pure compound **2** (cinnamaldehyde) against six kinds of citrus pathogens were shown in Figure 2. Cinnamaldehyde showed good antifungal activities against all of the tested citrus pathogens with the best against *Colletotrichum gloeosporioides* (Penz.). Cinnamaldehyde showed promising antifungal activities against citrus postharvest pathogens. *P. italicum* was the major pathogen causing the predominant citrus postharvest disease blue mold. *P. italicum* was selected to determine antifungal mechanism of cinnamaldehyde based on ^1^H-NMR metabolomics analysis.

### 2.4. Metabolites Identified in ^1^H-NMR Spectra

**Figure 3:**
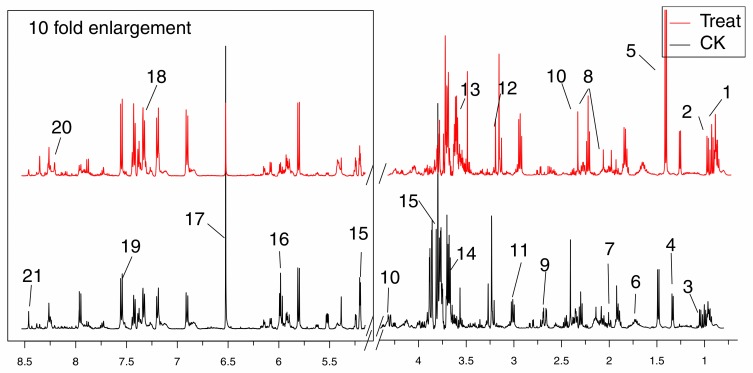
Typical 500 MHz ^1^H-NMR spectra obtained from the treat and CK groups. Metabolites were assigned: 1. Leucine; 2. Isoleucine; 3. Valine; 4. 2-Hydroxyisobutyrate; 5. Alanine; 6. Lysine; 7. Glutamate; 8. Methionine; 9. Malate; 10. Succinate; 11. Creatine; 12. O-Acetylcholine; 13. Glycine; 14. Mannitol; 15. Mannose; 16. UDP-glucose; 17. Fumaric acid; 18. Xanthosine; 19. Niacinamide; 20. Hypoxanthine; 21. AMP.

Representative 500 MHz ^1^H-NMR spectra were shown in Figure 3 with the assignment of metabolites. Resonances of metabolites were assigned by querying publicly accessible metabolomics databases such as Human Metabolome Database (HMDB, http://www.hmdb.ca) and Madison-Qingdao Metabolomics Consortium Database (MMCD, http://mmcd.nmrfam.wisc.edu), aided by Chenomx NMR suite 7.5 (Chenomx Inc., Edmonton, AB, Canada) and statistical total correlation spectroscopy (STOCSY) technique.

### 2.5. Multivariate Analysis of ^1^H-NMR Spectral Data

**Figure 4:**
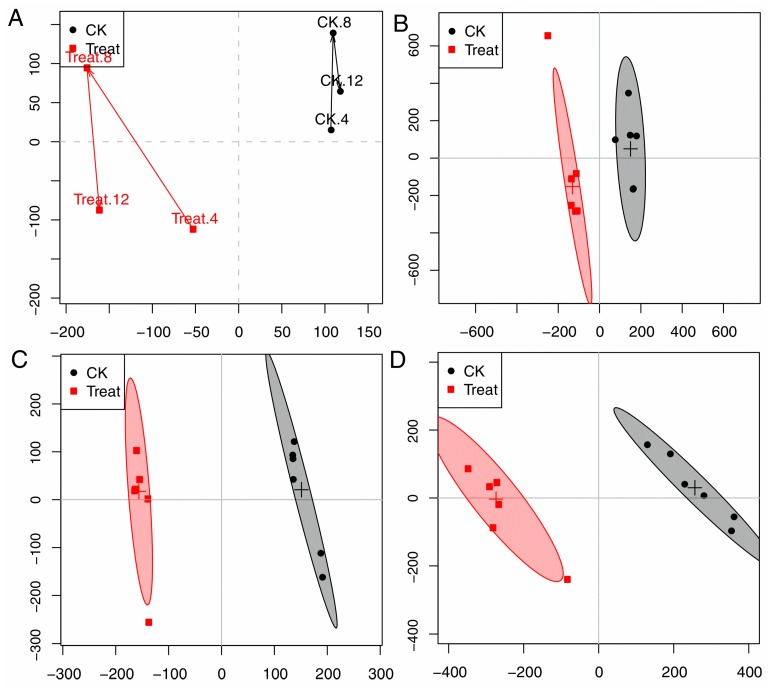
(**A**): Score trajectory plot from OSC-PLS-DA analysis of the CK and treatment groups (4, 8, 12 mean the time periods in hours); (**B**–**D**): Scores plot of CK and treatment groups in 4, 8, and 12 h, respectively.

In order to dynamically explore antifungal activities of compound **2** (cinnamaldehyde) against *P. italicum*, an OSC-PLS-DA model was constructed to analyze all the NMR data acquired from the control (CK) and treatment groups at 4, 8, and 12 h. The trajectory plot (Figure 4) exhibited a good separation between the CK and treatment group, showing an apparent time-dependent antifungal activities of cinnamaldehyde. The metabolomic changes in treatment group increased from 4 h to 12 h. A radical alteration in the metabolomic profiles of treatment groups happened at 12 h, reflecting a prompt response of the strain to cinnamaldehyde.

**Figure 5:**
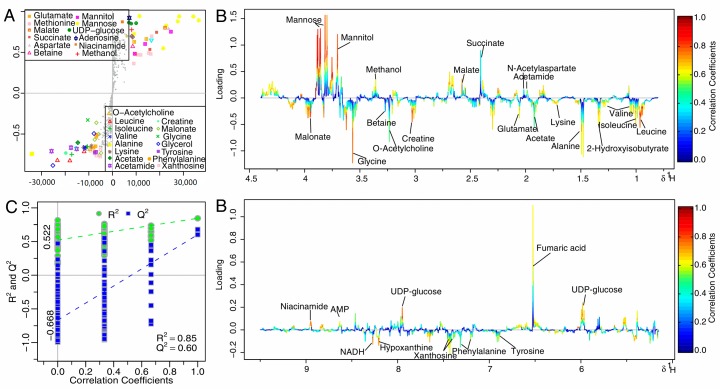
S-plot (**A**) and loadings plot with the metabolites labeled; (**B**) corresponding to the OSC-PLS-DA analysis from the CK and treatment groups at 12 h; (**C**) OSC-PLS-DA scatter plots of statistical validation obtained by 200 times permutation test, with R^2^ and Q^2^ values in the vertical axis, the correlation coefficients (between the permuted and true class) in the horizontal axis, and the ordinary least squares (OLS) line for the regression of R^2^ and Q^2^ on the correlation coefficients. Intercepts: R^2^ = (0.00, 0.522), Q^2^ = (0.00, −0.668).

The two groups were well separated from each other in the score plots (Figure 4A,C,E for 4, 8, 12 h, respectively), each point manifested a sample and each clustering represented a corresponding metabolic pattern in different groups. The CK and treatment groups were the furthest away in the scores plot at 12 h (Figure 4D), with class discrimination statistical parameters R^2^ 0.85 and Q^2^ 0.60 (Figure 5C), revealing prompt metabolic changes at 12 h. 

The S-plot is a scatter plot that visualize both the covariance (X axis) and correlation (Y axis) structure of loading profiles, thus would be helpful for filtering interesting metabolites in the projection, and for lowering the risk of false positive in metabolite selection. The loading plot was color-encoded according to the correlation coefficients (r^2^) and visualized in a covariance-based pseudo-spectrum in the OSC-PLS-DA model, with blue the least important metabolic changes and red the most important. The S-plot (Figure 5A) and loading plot (Figure 5B) revealed marked metabolic changes in extracts of the treatment group compared with the CK group: significantly decreased levels of acetamide, *N*-acetylaspartate, methionine, succinate, malate, methanol, mannitol, mannose, uracil, UDP-glucose, fumaric acid, AMP, and niacinamide; and markedly increased concentrations of leucine, valine, isoleucine, 2-Hydroxyisobutyrate, alanine, lysine, acetate, glutamate, methionine, creatine, O-acetylcholine, betaine, glycine, malonate, phenylalanine, tyrosine, xanthosine, NADH, phenylalanine, and hypoxanthine.

### 2.6. Metabolomics Analysis

**Figure 6:**
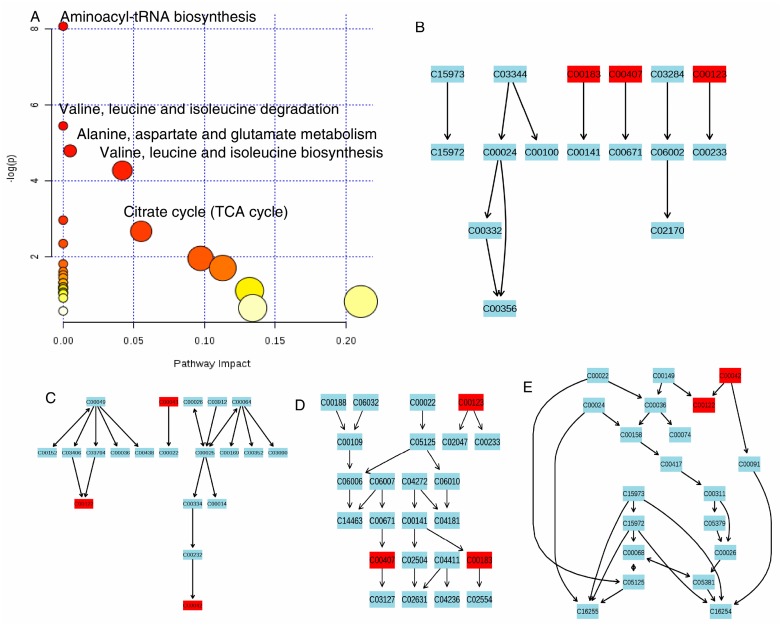
Pathway topology analysis associate with antifungal activities was carried out by MetaboAnalyst in this study (**A**); The term ‘‘log P” is the transformation of original *p*-value calculated from the enrichment analysis and the term ‘‘Impact’’ is the pathway impact value calculated from the pathway topology analysis. Bubble area is proportional to the impact of each pathway, with color denoting the significance from highest in red to lowest in white. Valine, leucine, and isoleucine degradation (**B**); Alanine, aspartate, glutamate metabolism (**C**); Valine, leucine, and isoleucine biosynthesis (**D**); citrate cycle (TCA cycle) (**E**).

**Figure 7:**
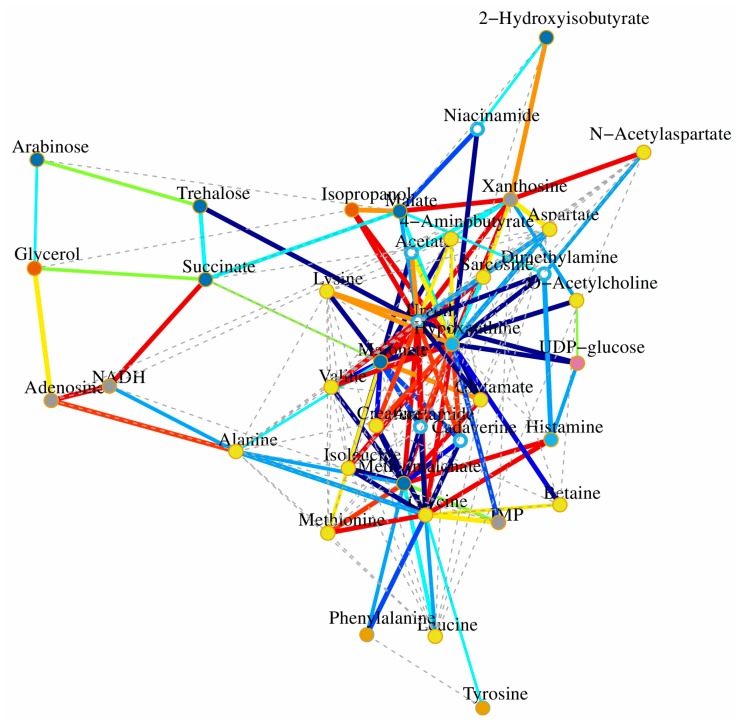
Correlation network determined by canonical (sparse-partial least squares, sPLS) analysis using metabolite concentrations as *X* variables and other parameters as *Y* variables. The network is graphically represented with metabolites and parameters as nodes, and correlations above a threshold (0.8) as edges (color coded according to the correlation coefficients, bluish for negative and reddish for positive correlations).

In this study, ^1^H-NMR based metabolomics approach was applied to comprehensively investigate antifungal activities of the pure compound **2** (cinnamaldehyde). Significant metabolites selected based on OSC-PLS-DA loading/S-plots were subjected to pathway analysis using MetPA (http://www.metaboanalyst.ca) (Figure 6A) and KEGG (http://www.genome.jp/kegg/) to identify biologically meaningful metabolic patterns and relevant pathways (Figure 6B–E). Canonical (sparse-partial least-squares, sPLS) analysis of the data was performed and graphical representation of the results (Figure 7) was generated using a web interface from the University of Queensland (http://mixomics.qfab.org) with metabolite concentrations as X variables and the other parameters as Y variables. ^1^H-NMR based metabolomics approach revealed metabolic changes induced by compound **2** that were related to disorders of energy metabolism and amino acids metabolism and oxidative stress.

Significant decrease of UDP-glucose was observed in treatment group as compared with CK group. UDP-glucose is an essential metabolite for a variety of processes in the cell physiology in all organisms. It is involved in the synthesis of trehalose (an osmoprotectant) and in galactose utilization [14].

Carbohydrates have been reported to increase production of secondary metabolites and proteins in fungi, occurring via an upregulation of fatty acid biosynthesis and an increase in the overall activity of the TCA cycle [15]. Decreased levels of mannose and its precursor (mannitol), and TCA cycle intermidiates (succinate, malate, fumaric acid) were observed in treatment group. TCA cycle is the most efficient and major source of energy supply, hampered activity of which brought about energy deficiency, so other means of ATP production have to come to the rescue. An acceleration of fatty acids β-oxidation to supply energy was indicated by the observed increase of acetate. In addition, decrease of creatine was found after cinnamaldehyde administration. Creatine kinase catalyzed the reaction of ADP with phosphocreatine to generate ATP to sustain ATP levels, especially under energy depletion.

Aminoacylation of tRNAs (shown in Figure 6) is critical step of protein biosynthesis [16]. Branched-chain amino acids (BCAAs, leucine, valine, isoleucine), aromatic amino acids (AAAs, phenylalanine, and tyrosine), alanine and lysine were essential precursors for protein synthesis and energy production. The great increase of BCAAs, AAAs, alanine, and lysine after cinnamaldehyde administration revealed increased degradation of proteins and energy metabolism disorder in *P. italicum* induced by cinnamaldehyde. In Figure 6, it also showed that valine, leucine, and isoleucine degradation and biosynthesis were imbalanced; and alanine, aspartate, and glutamate metabolism was upregulated. In addition, upregulation of phenylalanine and tyrosine metabolism after cinnamaldehyde administration was found, which has been similarly reported in mandarin orange after *R. paludigenum* immunity [17].

Significant decrease of *N*-acetylaspartate (NAA), a metabolite synthesized in mitochondria, was observed in treatment group. Considerable evidences show that NAA plays multiple roles in human brain, such as axon-glial signaling and metabolic function in mitochondria [18]. Significantly increased glutamate and glycine, precursors of glutathione, were observed in the treatment group, which showed the degradation of glutathione (GSH) by cinnamaldehyde in fungus. GSH functions as a reactive oxygen species (ROS) scavenger, and thereby protects the cell from free radical damage [19]. The potent anti-oxidant function of cinnamaldehyde in vivo supports further investigation for repurposing it for use as an antifungal agent. In addition, increased level of betaine (trimethylglycine), an organic osmolyte, could protect cells under stress and maintain the structural and functional integrity of cell membranes in treatment group, regarding as self-protective mechanism [20].

Significantly decreased concentrations of niacinamide; and markedly increased concentrations of xanthosine, hypoxanthine were observed in extracts of the treatment group compared with the CK group showed the effect of cinnamaldehyde on nucleic acid metabolism. Nucleobases, nucleosides, and their analogues can be exploited to design specific inhibitors for the microbial enzymes [21].

In conjunction with anti-microbial test, ^1^H-NMR-based metabolomics approach was firstly applied in this study to explore antifungal activities of compound **2** (cinnamaldehyde). Cinnamaldehyde could cause the abnormal metabolic state in *P. italicum* by interfering with different metabolic pathways such as disturbance in metabolism of amino acids, nucleic acids, and energy substances. Metabolomics showcased its ability to characterize the global metabolic status to evaluate the holistic antifungal activities of compound **2** (cinnamaldehyde). This study may promote the understanding of fungal infection and of the mechanism underlying the antifungal activities of cinnamaldehyde.

## 3. Materials and Methods

### 3.1. General Experimental Procedures

*Ramulus cinnamomi* (RC) were bought in June 2013 from Zhangshu medicinal market, Jiangxi Province, China, and authenticated by Prof Shouran Zhou (College of Basic Medicine, Jiangxi University of Traditional Chinese Medicine). A voucher specimen (no. RC-201306) was deposited in the herbarium of Jiangxi Key Laboratory for Postharvest Technology and Nondestructive Testing of Fruits & Vegetables, Jiangxi Agricultural University (Jiangxi, China).

*Penicillium italicum, Penicillium digitatum, Geotrichum candidum* Link, *Colletotrichum gloeosporioides* (Penz.), *Alternaria citri*, and *Phytophthora* spp. were provided by the Jiangxi Key Laboratory for Postharvest Technology and Nondestructive Testing of Fruits & Vegetables (Nanchang, China). All the test strains were preserved on potato dextrose agar.

^1^H- and ^13^C-NMR spectral data were tested on a Varian 400 MHz Nuclear magnetic resonance spectrometer. HPLC was conducted on a Hitachi Elite 2100 system, Luna C18 column (5 µm, 4.6 mm × 250 mm) for analysis and Luna C18 column (5 µm, 10 mm × 250 mm) for semi-preparative HPLC were purchased from Phenomenex Inc. The HPLC grade solvents were purchased from Sigma (Sigma, USA). All analytical solvents were bought from Tansoole (Shanghai, China).

### 3.2. Extraction and Isolation

The dried *Ramulus cinnamomi* (1.0 kg) were ground and extracted using ultrasonic-assisted method with 95 % ethanol (3 × 10 L) at 60 °C for 40 min. The extract were evaporated to remove ethanol solvent and yielded the dried ethanol extract (89.1 g), which was subjected to liquid–liquid extraction by ethyl acetate (RCA) and further on *n*-buthanol (RCB), respectively. 

The RCA showed better antifungal activities than RC extracts and RCB. As a result, the RCA was further isolated using silica gel column chromatography using CH_3_Cl–MeOH (100:1 to 10:1, *v*/*v*) for elution to yield four subfractions (RCA-1‒RCA-4). The RCA-1 fraction showed the best antifungal activities. RCA-1 fraction was purified by Sephadex LH-20 elution with MeOH and semi-preparative HPLC elution with MeOH–H_2_O to get compound **1** and compound **2.**

### 3.3. Antifungal Activity Tested by the Oxford Cup Method

The antifungal activities of RC extracts, ethyl acetate (RCA) and *n*-buthanol (RCB) fractions against *P. italicum* and *P. digitatum* were evaluated by the Oxford Cup method as described previously [10]. In short, Petri dishes (diameter, 9.0 cm) were prepared with PDA and surface inoculated with 2.0% of spore suspensions (10^5^–10^6^ CFU/mL) in sterile saline solution. Sterile Oxford cups (diameter, 8 mm) were impregnated with 200 μL of each extract. The diameters of inhibition zones (DIZs) around the Oxford cups were evaluated by Vernier micrometer after 48 h of culture at 27.0 ± 1.0 °C in the dark. Five replicate trials were conducted for each extract. The antifungal results were expressed as the mean value of diameters ± standard deviation. 

### 3.4. Antifungal Activity Tested by the Mycelial Growth Method

The antifungal activities of the pure compound **2** (cinnamaldehyde) against six kinds of citrus pathogens were examined by the mycelia growth method as described previously [10]. In short, the cinnamaldehyde were dissolved in sterile distilled water with 0.1% Tween 80, and then added to the sterile culture medium (PDA) at the specified concentrations. The mixed media were then poured into plastic Petri dishes (90 mm). The agar-mycelial plugs (6 mm) infected with different citrus pathogens were incubated at the center of the Petri dishes sealed with parafilm and incubated in the dark. Mycelium colony growth diameters were measured when the fungal mycelium of the control group had completely covered the Petri dishes. All treatments were tested in six replicates. The inhibition of mycelial growth (IMG, %) was calculated as the following formula: IMG (%) = 100 × (dc − dt)/(dc − 6), where dc and dt were the mycelium diameters (mm) of the control and the treatment, respectively.

### 3.5. ^1^H-NMR Based Metabolomics Analysis

^1^H-NMR based metabolomics analysis of the response of *P. italicum* to cinnamaldehyde was conducted. To 89 mL sterile culture medium (PDB), 1 mL of spore suspensions (10^8^ CFU/mL) were added, after two days incubation in shake (250 rpm/min), 10 mL cinnamaldehyde (1.0 mg/mL) was added to reach the final concentration of cinnamaldehyde at 0.1 mg/mL. The *P. italicum* with or without (control group) cinnamaldehyde were incubated in shake (250 rpm/min) for different time. After 4, 8 and 12 h incubation, the mycelium were washed by ultrapure water and quenched using a previously described method [22]. The mycelium were harvested via centrifugation at 6000× *g* for 6 min under 0 °C. The quenched cell pellets were re-suspended in 10 mL of cold PBS and were washed three times. The mixture was then centrifuged at 6000 g for 3 min under 0 °C and the resulting pellet was stored under −80 °C until extraction. Metabolites were extracted by 5 mL cold acetonitrile/water (1:1, *v*/*v*) after 6 min of sonication bathing in ice. The mixture was then centrifuged at 12,000× *g* for 8 min and the supernatants were freeze-dried, and then stored under −80 °C until NMR analysis. 

For ^1^H-NMR analysis, the lyophilized extracts were reconstituted with PBS (pH 7.4), with 99.8% D_2_O and 0.05% (*w*/*v*) sodium 3-(trimethylsilyl) propionate-2,2,3,3-*d*_4_ (TSP) for referencing purposes. The mixture solutions were vortexed and then centrifugated to afford the supernatant, which was transferred to NMR tube for NMR recording. ^1^H-NMR spectra of the samples were obtained at 25 °C on a Bruker AV 500 MHz spectrometer, using a nuclear Overhauser enhancement spectroscopy (NOESY) pulse sequence (relaxation delay-90°-μs-90°-tm-90°-acquire-FID) with a total spin-echo delay (2 nτ) of 10 ms to suppress the signals of proteins. ^1^H-NMR spectra were collected with 128 transients into 32,768 (32 K) data points over a spectral width of 10,000 Hz, with an acquisition time of 3.27 s and a relaxation delay of 3.0 s. The spectra were Fourier transformed by multiplication of the FIDs with an exponential weighting function corresponding to a line-broadening of 0.3 Hz.

### 3.6. Spectral Pre-Processing and Data Analysis

All ^1^H-NMR spectra were phased, baseline-corrected, and aligned using TopSpin software (version 2.1, Bruker, Billerica, MA, USA) with the TSP signal as a reference. The ^1^H-NMR spectra were automatically exported to ASCII files using MestReNova (Version 8.0.1, Mestrelab Research SL, Santiago de Compostela, Spain), which were then imported into “R” (http://cran.r-project.org/), and peak aligned with an in-house developed R-script. The one-dimensional (1D) spectra were automatically binned between 0.2 and 10 ppm using a dynamic adaptive binning approach for statistical analysis. The region of residual water and affected signals (4.65–5.25) was excluded, the remaining regions of each spectrum were normalized using probability quotient normalization (PQN) and subsequently mean-centered and pareto-scaled before further multivariate analysis.

Orthogonal signal correction partial least-squares discriminant analysis (OSC-PLS-DA) were applied to NMR data. All OSC-PLS-DA models were validated by a repeated two-fold cross-validation and permutation test (200 times); the validity of models against overfitting was assessed by the parameter *R*^2^*Y*, and the predictive ability was described by *Q*^2^*Y*. Color-coded loadings plot and S-plot were constructed to reveal variables that contributed to the group separation. The *p*-values obtained by permutation tests that were less than 0.05 indicated the significance of the established OSC-PLS-DA model at a 95% confidence level. The threshold for significance was *p* < 0.05 for all tests.

## 4. Conclusions

Two compounds identified as cinnamic acid (**1**) and cinnamaldehyde (**2**) were isolated from the antifungal fractions of *Ramulus cinnamomi*. Compound **2** (cinnamaldehyde) showed good antifungal activity against six kinds of citrus pathogens including *P. italicum*, *P. digitatum*, *G. candidum* Link, *C. gloeosporioides* (Penz.), *A. citri*, and *Phytophthora* spp. The antifungal mechanisms of cinnamaldehyde against *P. italicum* were explored by ^1^H-NMR metabolomics, which revealed the antifungal mechanisms maybe through effect on the metabolic pathways such as disturbance in metabolism of amino acids, nucleic acids, and energy substances.

## Abbreviations

NMR	Nuclear magnetic resonance
^1^H-NMR	^1^H nuclear magnetic resonance
^13^C-NMR	^13^C nuclear magnetic resonance
NOESY	Nuclear overhauser enhancement spectroscopy
RC	*Ramulus cinnamomi*
RCA	Extraction of *Ramulus cinnamomi* by ethyl acetate
RCB	Extraction *Ramulus cinnamomi* by *n*-buthanol
OSC-PLS-DA	Orthogonal signal correction partial least-squares discriminant analysis
DIZs	Diameters of inhibition zones
HMDB	Human Metabolome Database
MMCD	Madison-Qingdao Metabolomics Consortium Database
STOCSY	Statistical total correlation spectroscopy
sPLS	Sparse-partial least-squares
BCAAs	Branched-chain amino acids
AAAs	Aromatic amino acids
NAA	*N*-acetylaspartate
GSH	glutathione
ROS	Reactive oxygen species
HPLC	High Performance Liquid Chromatography
PDA	Potato Dextrose Agar
PDB	Potato Dextrose Broth
IMG	Inhibition of mycelial growth
CFU	Colony-Forming Units
PBS	Phosphate buffered solution
PQN	Probability quotient normalization
